# Recent advances in immunotherapy and its combination therapies for advanced melanoma: a review

**DOI:** 10.3389/fonc.2024.1400193

**Published:** 2024-07-16

**Authors:** Jiamin Xu, Shukun Mu, Yun Wang, Suchun Yu, Zhongming Wang

**Affiliations:** ^1^ School of Health Science and Engineering, University of Shanghai for Science and Technology, Shanghai, China; ^2^ Department of Radiation Oncology, Shidong Hospital, Yangpu District, Shidong Hospital Affiliated to University of Shanghai for Science and Technology, Shanghai, China; ^3^ Department of Pharmacy, Shidong Hospital, Yangpu District, Shidong Hospital Affiliated to University of Shanghai for Science and Technology, Shanghai, China

**Keywords:** melanoma, immune checkpoint inhibitors, radiotherapy, combination therapy, oncolytic virus

## Abstract

The incidence of melanoma is increasing year by year and is highly malignant, with a poor prognosis. Its treatment has always attracted much attention. Among the more clinically applied immunotherapies are immune checkpoint inhibitors, bispecific antibodies, cancer vaccines, adoptive cell transfer therapy, and oncolytic virotherapy. With the continuous development of technology and trials, in addition to immune monotherapy, combinations of immunotherapy and radiotherapy have shown surprising efficacy. In this article, we review the research progress of immune monotherapy and combination therapy for advanced melanoma, with the aim of providing new ideas for the treatment strategy for advanced melanoma.

## 1 Introduction

Melanoma is one of the most lethal and aggressive malignant tumors in the world. Its morbidity and mortality are increasing year by year, and the incidence of melanoma in men is slightly higher than that in women (1). Melanoma mostly occurs in the skin but also in mucous membranes, the uveal tract, and leptomeninges (2). Moreover, melanoma is prone to exhibit microsatellite, satellite, lymph node, or distant metastasis patterns to the lungs, brain, liver, and soft tissues and has a high local recurrence ability (3).

Immunotherapy is a treatment method that activates the human immune system and relies on its own immune function to cause cancer cells to die. For patients with melanoma that is surgically resectable, immunotherapy can serve as an adjuvant treatment to improve the prognosis. For patients with inoperable melanoma, immunotherapy can replace surgery, providing a new treatment direction (4, 5). Common clinical immunotherapies include immune checkpoint inhibitors, bispecific antibodies, cancer vaccines, adoptive cell therapy, and oncolytic virotherapy. This article reviews the clinical application of immunotherapy for advanced (unresectable or metastatic) melanoma, as well as the latest progress of combined radiotherapy.

## 2 Immune monotherapy

### 2.1 Immune checkpoint inhibitors

The immune checkpoint includes cytotoxic T lymphocyte-associated antigen-4 (CTLA-4), programmed death-1 (PD-1) and its ligand (PD-L1), and lymphocyte activation gene-3 (LAG-3). They negatively regulate CD8^+^ T cells, and cancer cells may make use of this pathway to downregulate T cell responses, thereby avoiding immune attacks (6). Immune checkpoint inhibitors (ICIs), on the other hand, activate immune cell activity by blocking immune checkpoint molecules, thereby achieving anti-tumor effects. There are many factors that affect the efficacy of ICIs, including tumor microenvironment (TME), age, diet, genetics, intestinal microbiota, antibiotics, and infusion time of ICIs (7, 8). Among them, a study found that injecting ICIs during the day (before 3 p.m.) may better integrate with the intrinsic circadian rhythm of the immune system to prolong the overall survival (OS) of patients (9). In addition, the efficacy of different ICIs varies, and with the deepening of research, drugs are constantly being updated and upgraded.

#### 2.1.1 CTLA-4 inhibitor

Ipilimumab is a monoclonal antibody that blocks the binding of CTLA-4 to its ligand on antigen-presenting cells. In addition to poor physical condition and elevated lactate dehydrogenase and C-reactive protein levels, endostatin and galactin-3-binding proteins may serve as prognostic biomarkers for advanced melanoma patients receiving ipilimumab treatment (10). When using ipilimumab monotherapy, the dosage has an impact on the efficacy of advanced melanoma. A study showed that 10 mg/kg of ipilimumab brings more significant survival benefits to patients than 3 mg/kg of ipilimumab, with median OS of 15.7 months and 11.5 months, respectively (p = 0.04), and 5-year OS rates of 25% and 19%, respectively. However, the incidence of level 3 or 4 treatment-related adverse events (AEs) is also higher (36% vs. 20%) (11). Besides clinical trials, the Ipi4 study showed similar efficacy in real-world advanced melanoma patients receiving an intravenous injection of 3 mg/kg of ipilimumab, with a median OS of 12.1 months and a 5-year OS rate of 20% (12). Although ipilimumab was the first ICI, with the continuous development of drugs, ipilimumab monotherapy is no longer the preferred first-line treatment for advanced melanoma.

#### 2.1.2 PD-1 inhibitor

Humanized IgG4 kappa monoclonal antibodies that target PD-1 are nivolumab, pembrolizumab, cetrelimab, toripalimab, and camrelizumab. Nivolumab proved to be a highly durable first-line therapy for advanced melanoma in the CheckMate 066 trial, with a median overall survival of 37.3 months, a 5-year OS of 43%, and a progression-free survival (PFS) rate of 32% (13). In 151 previously untreated patients with unresectable advanced melanoma, pembrolizumab demonstrated sustained anti-tumor activity and tolerability in the KEYNOTE-001 study, with a five-year OS rate of up to 41% and a median OS of 38.6 months (14). Pembrolizumab had a lower incidence of 3–4 grade adverse events than ipilimumab, according to the KEYNOTE–006 research, and it has higher clinical efficacy in treating advanced melanoma. The two patient groups had respective median OS of 32.7 months and 15.9 months (p = 0.00049), and respective median PSFs of 8.4 months and 3.4 months (p < 0.0001) (15). Furthermore, research indicated that patients who achieve early disease stabilization with pembrolizumab have a fair likelihood of long-term survival and a high chance of establishing a complete or partial remission with ongoing pembrolizumab treatment (16). In the LUC1001 study, the results of cetrelimab’s first treatment for advanced solid tumors were encouraging, with an overall response rate of 28% for melanoma patients and a median PFS of 5.4 months (17). Similarly, camrelizumab showed preliminary anti-tumor activity in the first treatment of advanced solid tumor patients in Australia (18). Moreover, in the subsequent Phase I study on patients with advanced melanoma in Asia, the efficacy of camrelizumab was similar to that of toripalimab in the POLARIS-01 study, with objective response rates (ORRs) of 15.2% and 17.3%, respectively (19, 20).

Prolgolimab is an IgG1 anti-PD-1 monoclonal antibody with Fc-silenced LALA (L234A/L235A) mutations. The MIRACULUM study was the first to evaluate the clinical efficacy of prolgolimab in advanced melanoma. The overall response rates of intravenous injections of 1 mg/kg prolgolimab (Group 1) and 3 mg/kg prolgolimab (Group 2) were 38.1% and 28.6%, respectively. Among them, the efficacy of primary skin melanoma patients in Group 1 was more significant, with ORR and median PFS of 48.9% and 8.84 months, respectively (21).

#### 2.1.3 PD-L1 inhibitor

Atezolizumab, avelumab, and durvalumab are anti-PD-L1 agents that are mostly used in combination with targeted agents for the treatment of advanced BRAF-mutant melanoma. In a phase I trial, atezolizumab was safe and well tolerated as a first-line monotherapy in patients with advanced BRAF-mutant melanoma, with an ORR and median PSF of 35% and 3.7 months, respectively (22). The international phase III trial IMspire170 found that atezolizumab in combination with cobimetinib (a MEK inhibitor) did not improve PSF in patients with advanced melanoma compared to pembrolizumab monotherapy, with 5.5 and 5.7 months, respectively (23). However, the IMspire150 trial showed that a triple combination with atezolizumab significantly prolonged PFS in patients with BRAF-mutated melanoma compared to a combination of vemurafenib (a BRAF inhibitor) and cobimetinib (15.1 months vs. 10.6 months, p = 0.025) (24). A phase I trial found durvalumab in combination with dabrafenib (a BRAF inhibitor) and trametinib (a MEK inhibitor) to be feasible for the treatment of BRAF-mutant melanoma (25). And avelumab showed a durable response in previously treated patients with metastatic melanoma, with an ORR of 21.6% and median PSF and OS of 3.1 and 17.2 months, respectively (26).

### 2.2 Bispecific antibodies

Bispecific antibodies have been extensively studied as an effective immunotherapy for advanced melanoma (**Table 1**).

**Table 1:** Clinical application of bispecific antibody in advanced tumors.

Researchers	Drug name	Tumor type	Clinical results
gp100 × CD3			
Nathan et al. (27)	Tebentafusp	Metastatic uveal melanoma	1-year OS:76%; Median OS: 21.7months (first-line treatment)
Carvajal et al. (28)	Tebentafusp	Metastatic uveal melanoma	1-year OS: 62%; Median OS:16.8 months (Received previous treatment)
CD73 × TGF-β			
Tolcher et al. (29)	Dalutrafusp alfa	Advanced solid tumors	ORR:4.8%; DCR:38.1%
CTLA-4 × PD-1			
Zhao et al. (30)	QL1706	Advanced solid tumors	ORR: 12.5%~33%
Ma et al. (31)	KN046	Advanced solid tumors	Median OS:16.6 months
Pang et al. (32)	Cadonilimab	Advanced solid tumors	ORR:33%

gp100, glycoprotein 100; CD, cluster of differentiation; TGF-β, transforming growth factor-β; CTLA-4, cytotoxic T lymphocyte-associated antigen-4; PD-1, programmed death-1; OS, overall survival; ORR, objective response rate; DCR, disease control rate.

Tebentafusp is a bispecific fusion protein composed of soluble high-affinity T cell receptors, targeting glycoprotein 100 (gp100) and cluster of differentiation (CD) 3 (33). In 2022, tebentafusp was approved by the Food and Drug Administration for the treatment of advanced uveal melanoma (34). For patients with metastatic uveal melanoma, tebentafusp as a first-line treatment has a 1-year OS rate of up to 73%, bringing significant survival benefits to patients (27). And for patients who have received previous treatment, tebentafusp also exhibits strong anti-tumor activity. By using a gradual dosing regimen (20 μg on Day 1, 30 μg on Day 8, 68 μg on Day 15, then injecting 68 μg intravenously once a week), the median OS was 16.8 months. Treatment-related AEs mostly occurred in the early stages of treatment, and the incidence and severity of AE decreased with repeated administration (28, 35). IMA401 is a T-cell antigen receptor bispecific drug targeting MAGEA4/8, co-developed by Bristol Myers Squibb and Immatics. The drug has demonstrated complete remission in a variety of *in vivo* tumor models, and the therapy is currently undergoing a Phase I trial in solid tumors in anticipation of the results being announced.

Dalutrafusp alfa is a bifunctional humanized antibody that targets both CD73-adenosine and transforming growth factor-β (TGF-β), and patients with advanced solid tumors have good tolerance to it (29). From this, it can be seen that dalutrafusp alfa has certain therapeutic potential for patients with advanced melanoma.

QL1706 is a bispecific antibody generated by MabPair technology that also targets CTLA-4 and PD-1. In a phase I trial targeting patients with advanced solid tumors (including advanced melanoma), QL1706 showed good anti-tumor efficacy and tolerance (30). Based on the promising preliminary efficacy of QL1706, it is worth further clinical research in advanced melanoma. KN046 is a novel bispecific antibody produced by Chinese hamster ovarian cells that can simultaneously block CTLA-4 and PD-1. In a multicenter phase I study, KN046 showed encouraging anti-tumor activity and controllable safety in the treatment of advanced solid cancer, and patients with high expression of CD8 and PD-1 had a longer median OS (31). However, the sample size of advanced melanoma is relatively small, and its efficacy needs to be verified in a large sample size. Cadonilimab is a symmetric tetravalent bispecific antibody against CTLA-4 and PD-1 with lower toxicity than the combination of CTLA-4 inhibitors and PD-1 inhibitors. It exhibits good clinical activity in advanced gastric esophageal cancer and metastatic cervical cancer (32). For advanced melanoma, there is still a lack of clinical trials to verify the efficacy of cadonilimab, but it is worth a try.

### 2.3 Cancer vaccines

Cancer vaccines are a promising immunotherapy consisting of tumor-related antigens and vaccine adjuvants. Common vaccines for treating melanoma include peptide vaccines, nucleic acid vaccines, cell vaccines, and nano vaccines.

Dendritic cell (DC) vaccines have been widely studied in melanoma. A team in the United States developed a DC-based genetically engineered vaccine using a recombinant adenovirus that co-expresses three melanoma antigens (tyrosinase, MART-1, and MAGE-A6). The study found that the expression of an inducible co-stimulator ligand on DCs in melanoma patients was positively correlated with the patient survival rate (36). This suggests that the treatment outcomes of patients can be improved by regulating the expression of an inducible co-stimulator ligand. The allogeneic plasmacytoid dendritic cells (PDCs) vaccine can initiate and amplify tumor-specific T cells. In the GeniusVac-Mel4 clinical trial, allogeneic PDC vaccines were subcutaneously injected into patients with metastatic melanoma, and the patients showed good tolerance (37). The tumor lysate particle-loaded dendritic cell vaccine is a personalized vaccine made by loading autologous tumor lysate into yeast cell wall particles and introducing patient DCs. Upregulation of genes related to DC maturation in tumor lysate particle-loaded dendritic cell vaccines that did not harvest DC using granulocyte colony stimulating factors can bring outstanding survival benefits to patients with advanced melanoma (3-year disease-free survival and OS rates of 55.8% and 94.2%, respectively) (38).

In addition to the DCs vaccine, there are other vaccines that exhibit anti-tumor activity against advanced melanoma. For example, vaccines developed for major histocompatibility complex-restricted phosphopeptides have been used for the first time in patients with advanced melanoma. Among them, the pIRS21097–1105 and pBCAR3126–134 phosphopeptide vaccines have good tolerance and clinical benefit prospects (39). A recombinant HSP110–gp100 vaccine was prepared from heat shock proteins 110 (HSP110) derived from autologous tumors and melanoma-associated antigen gp100. The lower dose (30–60 mcg) of the recombinant HSP110–gp100 vaccine showed clinical activity and low toxicity in patients with advanced melanoma (40). These vaccines are worth further research to improve the prognosis of patients.

### 2.4 Oncolytic virotherapy

Oncolytic virotherapy is a new type of immunotherapy. The use of natural or genetically recombinant viruses, through a dual mechanism of selective killing of tumor cells and inducing systemic anti-tumor immunity, brings clinical benefits to cancer patients (41). It has a good therapeutic effect in treating advanced melanoma (**Table 2**).

**Table 2:** Clinical application of Oncolytic virotherapy in the treatment of advanced melanoma.

Researchers	Drug name	Virus type	Patient characteristics	Clinical results
Andtbacka et al. (42)	T-VEC	Herpes simplex virus 1	Stage IIIB-IVM1c melanoma	Median OS:23.3months; ORR:31.5%; DCR:76.3%
Yamazaki et al. (43)	T-VEC	Herpes simplex virus 1	Stage IIIB–IVM1c Asian melanoma	Best Overall Response Rate:11.1%; DRR:11.1%; Median OS:22.87 months
Franke et al. (44)	T-VEC	Herpes simplex virus 1	Stage IIIB/C-IVM1a melanoma	Best Overall Response Rate:88.5%; DCR:92.3%
Cui et al. (45)	OrienX010	Herpes simplex virus 1	Stage IIIB/C–IVM1c Asian melanoma	ORR:19.2%; DCR:53.8%; Median OS:19.2 months
Kiyohara et al. (46)	GEN0101	Hemagglutinin virus of Japan	Stage IIIC-IV advanced metastatic melanoma	ORR:33.3%
Andtbacka et al. (47)	V937	Coxsackievirus	Stage IIIC-IVM1c melanoma	PFS:38.6%; DRR:21.1%
Beasley et al. (48)	PVSRIPO	Poliovirus 1	Stage IIIC–IVM1b anti-PD-1 refractory melanoma	50% patients remained without progression at a median follow-up time of 18 months

OS, overall survival; ORR, objective response rate; DCR, disease control rate; DRR, durable response rate; PFS, progression-free survival.

Talimogene laherparepvec (T-VEC) is a type 1 genetically modified herpes simplex virus. Through genetic modification, the deletion of the infected cell protein (ICP) 34.5 gene and the ICP47 gene, as well as the addition of granulocyte macrophage colony stimulating factor (GM-CSF), enhances the anti-tumor effect (49). Research has found that increasing the infection of T-VEC on susceptible tumor cells can improve the therapeutic effect of T-VEC, and overexpression of GM-CSF may have a negative impact on the curative effect of T-VEC. Therefore, the activation rate of GM-CSF is worth considering when designing T-VEC (50). For non-Asian populations, the OPTiM study found that compared to subcutaneous injection of GM-CSF, T-VEC had a more pronounced impact on OS in patients with advanced melanoma (23.3 months vs. 18.9 months, p = 0.0494), with ORRs of 31.5% and 6.4%, respectively, and a durable response rate (DRR) of 19.0% and 1.4%, respectively (42). For the Asian population, a multicenter Phase I trial in Japan showed that T-VEC exhibited similar clinical activity in patients with unresectable advanced melanoma, with a DRR of 11.1%. Among them, 77.8% of patients had treatment-related AEs, but their tolerance is good (43). For patients with early-stage metastatic melanoma, the optimal overall response rate of T-VEC is as high as 88.5%, and the disease control rate (DCR) is 92.3% (44). A clinical prediction model in the Netherlands further validated the above results, and patients with lower tumor burden showed better efficacy in using T-VEC (51).

OrienX010is also a type 1 herpes simplex virus that has undergone genetic modification. Its main operations include deleting the ICP34.5 gene and the ICP47 gene and inserting the inactivated ICP6 gene (which can reduce the potential neurotoxicity of injection) and GM-CSF. Intratumoral injection of OrienX010has a positive anti-tumor effect on Asian advanced melanoma patients, with an ORR of 19.2% and a DCR of 53.8% (45). And for patients with heavier tumor loads, higher doses of OrienX010may have more remarkable survival benefits.

The Hemagglutinin virus of Japan (HVJ) is a single-negative-stranded RNA virus. RNA fragments in the HVJ-envelope (HVJ-E) are absorbed into cells through membrane fusion, which can induce cancer cell death and stimulate anti-tumor immunity (52). In the Phase I clinical trial, HVJ-E (GEN0101) with 30000 mNAU and 60000 mNAU was administered to patients with advanced melanoma without dose-limiting toxicity. The anti-tumor activity increased in a dose-dependent manner, and the target lesion response rate increased from 44% in the low-dose group to 78% in the high-dose group (46).

Coxsackievirus A21 (V937) is a single-positive-stranded RNA enterovirus that can enter cells through overexpressed intercellular adhesion molecule-1 and decay-accelerating factor receptors in melanoma. The use of V937 in the CALM trial for the treatment of unresectable advanced melanoma resulted in a 6-month PFS rate of 38.6% and a DRR of 21.1%, with good patient tolerance and no treatment-related AEs of grade 3 or above (47).

PVSRIPO is a genetically modified type 1 poliovirus that can infect CD155-expressing antigen-presenting cells, thereby triggering an immune response targeting tumors. For advanced melanoma patients who are difficult to treat with PD-1, injection of PVSRIPO into the tumor shows clinical activity and good tolerance (48).

### 2.5 Adoptive cell therapy

Adoptive cell therapy refers to the separation of immune-active cells from tumor patients, followed by *in vitro* culture, amplification, and functional identification before re-infusion into the patient’s body, achieving the goal of directly killing the tumor or activating the immune response in the body to eliminate the tumor (53). Immune cells mainly include tumor-infiltrating lymphocytes (TILs), natural killer cells, lymphokine-activated killer cells, etc. TIL therapy has been extensively studied in advanced melanoma and usually requires a combination of immune regulatory factors to stimulate TIL activity. Interleukin-2 (IL-2) is a cytokine stimulator for T cells, natural killer cells, lymphokine-activated killer cells, and B cells, which can promote lymphocyte growth, proliferation, and differentiation and is often used in combination with TIL.

In a phase III multicenter trial (NCT02278887) in the Netherlands, the median PFS of 84 patients receiving TIL (intravenous high-dose IL-2) treatment for unresectable advanced melanoma was significantly higher than that of ipilimumab treatment (7.2 months vs. 3.1 months, p < 0.001), but the incidence of treatment-related AEs also increased (54). However, in a phase II trial, subcutaneous injections of low-dose IL-2 (125000 IU/kg/day) were administered to patients with advanced melanoma using TIL therapy. Although reducing the toxicity caused by IL-2, the curative effect also decreased (55). For advanced melanoma patients who have failed ICIs treatment, TIL therapy (intravenous high-dose IL-2) has shown clinical activity in different subtypes (mucosal, superficial diffusion, and limb), but there are reversible AEs of grade 3 and grade 4 (56). In addition, there are also studies using low-dose interferon alpha to replace the previous pretreatment process (nonmyeloablative lymphodepleting chemotherapy combined with high-dose IL-2) for TIL therapy. The clinical benefits to patients are long-lasting, with a 3-year OS of 46.7% in responsive patients. Nevertheless, interferon alpha can lead to a decrease in white blood cells, neutrophils, and lymphocytes (57). Lifileucel (LN-144), a TIL therapy developed by Iovance Biotherapeutics, is produced from harvested tumor specimens using a simplified 22-day process in a centralized Good Manufacturing Practices facility, and in the Phase 2 C-144–01 trial, lifileucel demonstrated sustained clinical activity in the treatment of patients with advanced melanoma who had received prior ICI therapy (58). The therapy is currently in the FDA review stage and is expected to be the world’s first immunocellular therapy for the treatment of solid tumors (59).

In addition to using TIL for adoptive cell therapy, the MELSORT trial selected T lymphocytes with specificity for Melan-A and MELOE-1 antigens and high-dose IL-2. This combination has shown clinical activity in advanced melanoma patients and is expected to be used in conjunction with ICIs to enhance efficacy (60). IMA203 is an autologous T-cell receptor-engineered T-cell therapy developed on Immatics’ proprietary ACTengine platform targeting the melanoma antigen PRAME presented by HLA-A*02. The company has now announced clinical data from its ongoing 1b-dose expansion cohort A. The data showed that IMA203 was well tolerated and resulted in confirmed objective remissions in melanoma. The trial of IMA203 in combination with ICIs is ongoing, and the results are expected to be announced.

## 3 Dual immunotherapy

### 3.1 Dual ICIs therapy

The combination of CTLA-4 inhibitors and PD-1 inhibitors has shown more lasting clinical benefits than single therapy in unresectable advanced melanoma. In the Phase III CheckMate 067 trial (NCT01844505), the 5-year OS rate of ipilimumab combined with nivolumab for advanced skin melanoma was 52%, with a median PFS of 11.5 months and an ORR of 58%. 59% of patients experienced grade 3 or 4 treatment-related AEs (61). Wolchok et al. (62) further studied on the basis of this experiment and found that the 6.5-year median OS was 72.1 months, and the median melanoma-specific survival period was not reached at 77 months. The drug tolerance was good, and no new AE occurred. In addition to clinical trials, the combination of ipilimumab and nivolumab also brings long-term survival benefits to patients in the real world, with a 4-year OS of 50% for patients similar to the CheckMate 067 trial (63). For patients with metastatic uveal melanoma, ipilimumab combined with nivolumab as first-line treatment also showed outstanding clinical activity, significantly improving the patient’s OS compared to monotherapy (64, 65). In addition, the dosage of ICIs has a certain impact on anti-tumor activity and safety. The KEYNOTE-029 study showed that, compared with standard-dose ipilimumab (3 mg/kg) plus reduced-dose nivolumab (1 mg/kg) in the treatment of advanced melanoma, the combination of reduced-dose ipilimumab (1 mg/kg) and standard-dose pembrolizumab (2 mg/kg) significantly reduced the toxicity associated with the combination therapy and provided survival benefits to patients (66). This dose combination mode is also applicable to advanced melanoma patients who have failed PD-1 inhibitor treatment, with a median OS of 24.7 months and controllable safety (67).

The combination of LAG-3 inhibitors and PD-1 inhibitors is also more effective than monotherapy in the treatment of advanced melanoma and has the potential to become a first-line treatment. Relatlimab is a humanized monoclonal antibody targeting LAG-3. In the RELATIVITY-047 trial, a median PFS of 10.1 months was observed in untreated patients with advanced melanoma who received a single intravenous infusion of relatlimab (160 mg) and nivolumab (480 mg), which was significantly prolonged by 5.5 months compared to nivolumab monotherapy (p = 0.006). Although the incidence of AE was slightly higher, it was well tolerated (68). Schadendorf et al. (69) further demonstrated the above results by studying the health-related quality of life of patients based on this experiment. For advanced melanoma patients who have progressed after receiving previous treatment (including anti-PD-1 therapy), relatlimab combined with nivolumab can also bring long-lasting survival benefits. And Opdualag, a fixed-dose combination of relatlimab and nivolumab, has been approved by the FDA in March 2022 for the treatment of unresectable or metastatic melanoma (70). Efftilagimod alpha is a soluble LAG-3 fusion protein that can activate antigen-presenting cells, thereby activating CD8^+^ T cells. In a multicenter Phase I trial, for the first time, efftilagimod alpha combined with pembrolizumab was used to treat advanced melanoma. The treatment mode showed strong anti-tumor activity in both pembrolizumab refractory and pembrolizumab initially treated patients, with overall remission rates of 33% and 50% and DCR of 55.6% and 83%, respectively (71).

### 3.2 Cancer vaccine combined with ICIs

UV1 is a human telomerase reverse transcriptase vaccine composed of three synthetic long peptides, which can enhance anti-tumor activity when combined with ICIs. In the phase I/II single-center clinical trial (NCT02275416), the combination of UV1 and ipilimumab showed controllable safety in the treatment of metastatic melanoma with no additional toxicity. The ORR was 33%, the 5-year OS was 50%, and the median PFS was 6.7 months (72, 73). In addition, a multicenter phase I trial (NCT03538314) is underway for the treatment of advanced melanoma using UV1 combined with pembrolizumab.

Six melanoma helper peptide vaccines, limited by the class II major histocompatibility complex, can induce CD4^+^ T helper cell responses. Combined with pembrolizumab, they can increase the number of T cells, B cells, and Th1 cells in the tumor. Especially for advanced melanoma patients who did not receive other immunotherapies before the combination therapy, the clinical benefits were more significant (74).

The vaccine developed for the tumor associated-antigen NY-ESO-1 can induce CD4^+^ and CD8^+^ T cells, as well as antibody responses. Ipilimumab can also enhance the immunity of the NY-ESO-1 vaccine. The combination of the two can have a beneficial effect on TME in the treatment of advanced melanoma, increasing the proliferation of CD8^+^ T cells within the tumor and ensuring controllable safety (75).

NEO-PV-01 is a personalized vaccine made based on new antigens generated by cancer cell mutations. Its induced T cells have a cytotoxic phenotype and can be transported to tumors and kill cancer cells. The combination of NEO-PV-01 and nivolumab has shown strong potential in the treatment of advanced melanoma. In a phase Ι clinical trial, the ORR of patients after combination therapy was 59%, the 1-year OS rate was 96%, and there were no severe treatment-related AEs (76).

The phase 2 KEYNOTE-942 trial evaluated the efficacy of mRNA-4157/V940, a personalized cancer vaccine developed by Moderna, in combination with pembrolizumab for patients with advanced melanoma. The results showed a significant improvement in recurrence-free survival compared to pembrolizumab monotherapy, resulting in a 44% reduction in the risk of postoperative recurrence or death (77). These findings are encouraging and have led to the therapy being granted breakthrough therapy designation by the FDA.

### 3.3 Adoptive cell therapy combined with ICIs

A study has found that new epitope-specific CD8^+^ T cells affect the efficacy of TIL adoptive cell therapy in treating melanoma (78). Using ICIs to increase the frequency of CD8^+^ T cells may improve clinical outcomes. In a phase I trial, TIL adoptive cell therapy combined with nivolumab was safe and feasible for the treatment of metastatic melanoma patients who had not received PD-1 inhibitors, with an ORR of 36% and a median OS of 23 months. The use of CD137 agonists during TIL generation *in vitro* can reduce culture time and increase tumor-specific activity (79). TILVANCE-301 is a multicenter Phase 3 trial designed to evaluate the efficacy and safety of lifileucel in combination with pembrolizumab versus pembrolizumab alone in patients with untreated advanced melanoma. The trial is currently ongoing, and the results are promising.

### 3.4 Oncolytic virotherapy combined with ICIs

T-VEC can overcome adaptive resistance by inducing systemic immune activity and altering TME, and its combination with ICIs has shown excellent clinical efficacy in the treatment of advanced melanoma (80). A multicenter phase II trial with a 5-year follow-up showed that the ORR of T-VEC combined with ipilimumab for unresectable stage IIIB-IV melanoma was more than twice that of ipilimumab monotherapy (35.7% vs. 16.0%, p = 0.0003), with DRRs of 33.7% and 13.0%, respectively, and no additional toxicity (81). T-VEC in combination with pembrolizumab showed some improvement in efficacy in patients with advanced melanoma, with an ORR of 48.6% and a DRR of 42.2% (82).

The combination of V937 and ICIs has shown encouraging results in the treatment of advanced melanoma. The ORR of advanced melanoma patients in the CAPRA study receiving intratumoral injection of V937 combined with pembrolizumab treatment was 47%, with a median PFS of 11.9 months, both higher than monotherapy, and the safety was controllable (83). In the MITCI trial, the ORR and DRR of intratumoral injections of V937 and ipilimumab for advanced melanoma patients were 30% and 14%, respectively. Among them, patients who had previously undergone PD-1 inhibitor treatment and experienced disease progression also had clinical benefits (median OS of 29.7 months) (84). In addition to intratumoral injection of V937, the STORM trial showed that intravenous injection of V937 in combination with pembrolizumab has controllable safety in patients with advanced solid tumors (85). However, in the CLEVER study, intravenous injection of V937 combined with ipilimumab did not bring significant survival benefits to patients with advanced uveal melanoma (86).

RP1, a herpes simplex virus-based lysosomal viral therapy developed by Replimune, showed activity in combination with nivolumab in melanoma that failed PD-1 immunotherapy in the Phase 2 IGNYTE trial (NCT03767348), promising to provide patients with a novel treatment option. In addition to this, the team has developed RP2 (adding CTLA-4 antibody-like molecules to RP1) and RP3 (adding 4–1BBL and CD40L to RP2) and launched clinical trials in solid tumors.

### 3.5 Other immunologic combination therapies

For melanoma patients with ICI resistance (primary or secondary resistance), in addition to the aforementioned immune combination therapy, new therapies are also needed to improve the condition. The DC vaccine can effectively activate T lymphocytes, and its combination with TIL therapy may improve patient prognosis. In a phase I trial, this combination therapy showed clinical activity (87). Furthermore, the feasibility of DC plus TIL was further demonstrated in the Phase II trial, with a patient’s ORR of 50% (88). However, only DC vaccines targeting multiple antigens have shown efficacy, and both trials have small sample sizes, requiring further validation in more patients. In addition to being used in combination with TIL, the DC vaccines can also be combined with T-VEC. In a phase I trial, T-VEC and CD1c (BDCA-1) ^+^ bone marrow DC vaccine +/- CD141 (BDCA-3) ^+^ bone marrow DC vaccine were injected into advanced melanoma patients who failed ICIs treatment. The results were satisfactory, with 66.7% of patients (n = 3) experiencing persistent complete remission (89).

## 4 Immunotherapy combined with radiotherapy

### 4.1 Joint mechanism

During the occurrence and development of tumors, TME interacts with tumor cells, jointly mediating the immune tolerance of tumors, thereby affecting the therapeutic effect of immunotherapy. Radiotherapy is the use of energy emitted by radiation to cause structural changes and loss of biological activity in tumor cells, leading to tumor apoptosis and necrosis, thus achieving the goal of treatment. Research has shown that radiotherapy can induce immunogenic cell death, leading to a large number of damages-related molecular patterns and cytokine expression and release, activating anti-tumor immune responses (90). In addition, radiation therapy can also promote the infiltration of effector immune cells into TME, thereby enhancing the immune system’s ability to recognize and kill tumor cells (91, 92). And in some cases, local radiotherapy may also trigger a systemic anti-tumor response (distant effect) in unirradiated areas, which may be related to immune activation triggered by DNA damage induced by radiotherapy (93). Therefore, radiotherapy may enhance the effectiveness of immunotherapy and improve the prognosis of patients.

### 4.2 Clinical researches

The combination of immunotherapy and radiotherapy has great potential in the treatment of advanced melanoma (**Table 3**).

**Table 3:** Clinical application of immunotherapy combined with radiotherapy in advanced melanoma.

Researchers	Immunotherapy	Radiotherapy	Clinical results
Maity et al. (94)	3 mg/kg ipilimumab	HFRT	Median PFS:3.6 months; Median OS:10.7 months
Boutros et al. (95)	10 mg/kg ipilimumab	HFRT	Median PFS: 4.8 months; Median OS:10.8 months
Sundahl et al. (96)	Nivolumab	SBRT	Overall response rate: 45%
Curti et al. (97)	600000 IU/kg Interleukin-2	SBRT	ORR: 54%; DCR: 75%
Minor et al. (98)	Ipilimumab and nivolumab	Yttrium 90	ORR: 20%; DCR: 68%; Median PFS: 5.5 months; Median OS: 15.0 months
Cavalieri et al. (99)	Immune checkpoint inhibitors	Carbon iON radiotherapy	The experiment is currently underway

OS, overall survival; ORR, objective response rate; DCR, disease control rate; DRR, durable response rate; PFS, progression-free survival; HFRT, hypofractionated radiation therapy; SBRT, stereotactic body radiotherapy.

Hypofractionated radiation therapy combined with ipilimumab has also shown clinical activity in the treatment of metastatic melanoma. The median PSF and OS of 3 mg/kg ipilimumab combined with hypofractionated radiation therapy were 3.6 months and 10.7 months, respectively, and there was no dose-limiting toxicity caused by the combination therapy (94). The median PSF and OS of 10 mg/kg ipilimumab combined with hypofractionated radiation therapy were 4.8 months and 10.8 months, respectively (95). Although the doses of ipilimumab in the two Phase I trials mentioned above were different and the radiotherapy regimen was also different, the clinical results were similar.

Studies have found that stereotactic body radiation therapy (SBRT) combined with immunotherapy can alter the immune phenotype of blood T cells in patients with advanced melanoma (100). In a phase II trial, the overall response rate of SBRT combined with nivolumab for advanced melanoma was 45%, and it was found through circulating tumor DNA that the combination of SBRT can bring survival benefits to patients with lower PD-1 comprehensive positive scores (96). In addition, the combination of SBRT and high-dose IL-2 (600000 IU/kg) also showed satisfactory results, with ORR and DCR of 54% and 75%, respectively, in patients with metastatic melanoma (97). And the immune activity of this combination was further confirmed in a phase II experiment (101).

In a multicenter prospective clinical trial, patients with uveal melanoma with liver metastasis were treated with sequential hepatic arterial infusion of Yttrium 90 resin microspheres with a normal liver radiation limit dose of 35 Gy and combined with ipilimumab (1 mg/kg) and nivolumab (1 mg/kg or 3 mg/kg). The results of the study were promising. The ORR and DCR of patients were 20% and 68%, respectively. The median PFS and median OS are 5.5 and 15.0 months, respectively, and the toxicity is tolerable (98).

The ICONIC study is the first to combine carbon-ion radiation therapy with ICIs for the treatment of advanced solid tumor patients. The trial is currently underway, and its results may provide new treatment options for advanced melanoma patients (99).

## 5 Conclusion

Tumors evade the immune system through a synergistic process of central and peripheral tolerance. ICIs can overcome peripheral tolerance and bring survival benefits to cancer patients. However, for immunocompromised patients, long-term benefits cannot be achieved due to insufficient tumor-specific CD8^+^ T cells or insufficient new antigenicity. In addition to ICIs, bispecific immunotherapy, cancer vaccines, adoptive cell therapy, and oncolytic virotherapy are all immunotherapies that are currently being studied in advanced melanoma. The combination of different immunotherapies has brought new hope to patients who have not received immunotherapy or who have previously received immunotherapy but have progressed, and it has greatly improved the prognosis of patients with advanced melanoma. Based on the synergy between radiotherapy and immunotherapy, the combination of the two is promising. Further studies are needed on the selection of immunotherapy, dosage, and route of administration, as well as the choice of radiotherapy technique and radiation dose in combination therapy, in order to improve the survival rate of patients without adding additional toxic reactions.

## Author contributions

JX: Writing – original draft, Writing – review & editing. SM: Writing – review & editing. YW: Writing – review & editing. SY: Writing – review & editing. ZW: Writing – review & editing.
